# Use of task-shifting to scale-up child mental health services in low-resource Ugandan schools: role of contextual factors on program implementation

**DOI:** 10.1186/1748-5908-10-S1-A23

**Published:** 2015-08-20

**Authors:** Keng-Yen Huang, Janet Nakigudde, Laurie Miller Brotman

**Affiliations:** 1 Department of Population Health, New York University School of Medicine, New York, NY 10016, USA; 2 Department of Psychiatry, College of Health Sciences, Makerere University, Kampala, Uganda

## Objectives

Children in Sub-Saharan Africa (SSA) are burdened by significant unmet mental health needs, but this region has limited access to mental health services to address these needs. Despite the success of numerous interventions for promoting child mental health, most evidence-based programs (EBPs) are not available in SSA. This study utilizes a task-shifting strategy and trains teachers as community health workers (CHWs) to utilize EBP strategies and provide basic child mental health services in schools. This paper focuses on two implementation objectives: 1) investigating the transportability of an EBP (ParentCorps-a school-based mental health program) from a developed country to a SSA country-Uganda by evaluating quality of implementation; and 2) studying the influences of contextual factors (i.e., agency setting, individual characteristics) on implementation processes and outcomes (e.g., CHWs' level of engagement and utilization of EBP strategies).

## Methodology

The study is guided by two implementation frameworks-Consolidated Framework for Implementation Research and Teacher Training Implementation Model. A cluster randomized wait-list control design and a mixed method data collection method were applied. Eighty teachers from ten schools in Kampala Uganda were recruited.

## Results

Preliminary findings support the feasibility of transporting the EBP to developing countries. An EBP with a well structural implementation manual can be easily adopted and implemented with high fidelity in developing countries. Consistent with the hypothesis, contextual factors have important impacts on program implementation. Specifically, we found schools with more open communication climate predict better teachers' participation/engagement during training and higher frequency in utilizing EBP strategies.

## Implications to dissemination and implementation (D&I) research

This study addresses important D&I research gaps by providing evidence to support EBP transportability from a developed country to a low-income country and systematically studying factors that may contribute to effective task shifting.

